# Chronic idiopathic intestinal pseudo-obstruction treated with jejunostomy: case report and literature review

**DOI:** 10.1590/S1516-31802007000600011

**Published:** 2007-11-01

**Authors:** Carlos Renato dos Reis Lemos, Pedro Popoutchi, Rogério Serafim Parra, Omar Féres, José Joaquim Ribeiro da Rocha

**Affiliations:** Discipline of Proctology, Department of Surgery and Anatomy, Faculdade de Medicina de Ribeirão Preto (FMRP), Universidade de São Paulo (USP), Ribeirão Preto, São Paulo, Brazil

**Keywords:** Intestinal pseudo-obstruction, Intestinal obstruction, Jejunostomy, Parenteral nutrition, Diagnosis, Pseudo-obstrução intestinal, Obstrução intestinal, Jejunostomia, Nutrição parenteral, Diagnóstico

## Abstract

**CONTEXT:**

Chronic idiopathic intestinal pseudo-obstruction is a very rare condition.

**CASE REPORT:**

This study describes a male patient who had presented obstructive symptoms for 24 years. He had been treated clinically and had undergone two previous operations in different services, with no clinical improvement or correct diagnosis. He was diagnosed with intestinal obstruction without mechanical factors in our service and underwent jejunostomy, which had a significant decompressive effect. The patient was able to gain weight and presented improvements in laboratory tests. Jejunostomy is a relatively simple surgical procedure that is considered palliative but, in this case, it was resolutive.

## RESUMEN

**CONTEXTO:**

A pseudo-obstrução intestinal crônica idiopática é uma causa rara de obstrução intestinal.

**RELATO DE CASO:**

O presente estudo relata o caso de um paciente com queixas obstrutivas há longa data (24 anos); sendo já submetido a tratamentos clínicos e a duas laparotomias em outro serviço, não houve melhora dos sintomas e nem elucidação do diagnóstico. Foi diagnosticada obstrução intestinal sem fator mecânico e o paciente foi submetido a jejunostomia em nosso serviço, tendo apresentado importante efeito descompressivo. Houve ganho de peso ponderal e melhora nos exames laboratoriais. A jejunostomia é um procedimento cirúrgico relativamente simples, considerada paliativa, mas nesse caso, teve caráter definitivo e resolutivo.

## INTRODUCTION

Chronic idiopathic intestinal pseudo-obstruction (CIIPO) is a syndrome characterized by signs and symptoms of intestinal obstruction in the absence of mechanical factors, associated diseases or medicine intake.^1-3^ The usual treatment consists of prokinetic drugs, while severe cases are treated with enterostomies for enteral nutrition and decompression.^4^ The present study reports a case of CIIPO that was treated successfully by means of a venting jejunostomy and reviews the specific literature.

## CASE REPORT

A 60-year-old white male patient had been complaining of abdominal distension and discomfort episodes for 24 years. The first episode had occurred 24 years before the time the patient came to us, with abdominal distension, weakness, bilious vomiting and severe diarrhea, which had been treated clinically with only partial relief of the symptoms. Subsequently, similar episodes had occurred at a frequency of four times a year or less. The patient had undergone two operations: one in 1988, in which congenital bridle was diagnosed, and another in 2001, in which intestinal distension due to adhesions of the ileum was diagnosed. No intestinal resection was reported and there was no clinical relief of the symptoms after either of these operations.

The patient was seen in our service in 2001 with the same symptoms as before. No familial history of multiple incidence diseases was reported and the patient had no recognized underlying disease. The patient underwent the following tests: Chagas disease serology and thyroid function, which were normal; and gastric-emptying scintigraphy, digestive endoscopy, intestinal-transit time and colon-transit time, which presented diffuse dilation of the small bowel, megaduodenum and colonic inertia. Radiographic examination showed diffuse intestinal dilatation, with megaduodenum that was compatible with intestinal pseudo-obstruction and colon palsy. A biopsy was performed on the biceps brachii muscle, and the result from this was suggestive of denervation associated with slight mitochondrial dysfunction, thus corroborating the hypothesis of CIIPO.

Clinical treatment with prokinetic drugs (cisapride and metoclopramide) and octreotide had no success. Exploratory laparotomy was performed, which showed diffuse small intestine and right colon dilatation, with no mechanical obstruction. An enteric biopsy and a Witzel venting jejunostomy were performed. The biopsy result was inconclusive. As an incidental finding, urinary bladder pseudo-diverticula were found during urethrocystoscopy that was performed because of urine retention after removing the bladder catheter.

The patient tolerated the postoperative period and reported relief of the symptoms and good acceptance of the oral diet. The jejunostomy had a very significant decompressive effect. Over the course of the follow-up, the patient has been accepting a regular oral diet, with no vomiting or abdominal pain. His intestinal habit is regular, three to four times a day, with semi-solid feces. The jejunostomy debit is approximately 1000 ml per day. The patient has gained weight and has shown significant improvement in laboratory tests (Table 1). The patient's present weight is 63 kg.

**Table 1:** Laboratory tests and weight before and after venting jejunostomy in a case of chronic idiopathic intestinal pseudo-obstruction

Exams	July 2001	September 2003
Albumin (g/dl)	2.9	3.6
Total proteins (g/dl)	5.6	6.8
Aspartate aminotransferase (u/l)	158.7	16
Alanine aminotransferase (u/l)	299.5	29
Alkaline phosphatase (u/l)	131.6	60
Weight (kg)	51	63

## DISCUSSION

CIIPO is a syndrome of ineffective motility due to primary dysfunction of the enteric nerve or muscle, and it is rare.^5^ The first finding is usually clinical and radiological evidence of intestinal obstruction without any mechanical injury. This condition was first recognized by Dudley et al.,^2^ who described 13 patients with clinical signs of intestinal obstruction with no mechanical cause, who underwent multiple laparotomies because of recurrent symptoms of abdominal pain, distension and vomiting. The term "chronic idiopathic intestinal pseudo-obstruction" was first used by Maldonado et al.,^3^ who described five patients with recurrent unexplained episodes of intestinal obstruction, associated with diarrhea and weight loss. Some of their patients progressed to starvation and death.

**Figure 1:**
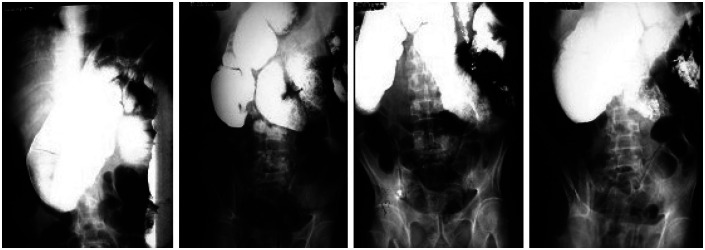
Radiographic examination suggesting dilatation without stenosis in a case of chronic idiopathic intestinal pseudo-obstruction.

Many patients present symptoms for several years before undergoing laparotomy, because recognition of their abnormal intestinal function is frequently delayed. The most common misdiagnosis in CIIPO cases is intestinal obstruction caused by the superior mesenteric artery. Other diagnoses prior to identification of CIIPO include achalasia, diverticulosis, psychogenic vomiting, functional bowel disease, megacolon, colitis, megaduodenum, malabsorption syndrome and adhesions. Abdominal pain and vomiting are the most common symptoms.

CIIPO usually has a long and progressive course that is characterized by relapses,^1^ and the literature suggests that the pharmacological response to this primary motility disorder is poor.^2^ On the other hand, surgical treatment is usually avoided in the absence of mechanical obstruction. Nevertheless, some patients will benefit from palliative surgery when the aim is to relief specific symptoms. Choosing which patients will benefit from surgical procedures has to be done through very careful analysis of the gastrointestinal radiographic examinations, together with assessment of the severity of symptoms. If anatomical or functional abnormalities are identified, and are related to the clinical symptoms, palliative surgery to correct the abnormalities will succeed.^3^

Surgical or endoscopic gastrostomy or jejunostomy may be required for CIIPO patient nutrition. Chronic vomiting, abdominal distension and pain can be treated by means of venting gastrostomy or enterostomies, and these procedures have been described as successful. Such procedures shorten the length of hospital stay and avoid unnecessary laparotomy.

Definitive treatment for CIIPO is only possible through small intestine transplantation. This is a new procedure that is not free of risks and is indicated for patients with CIIPO associated with intestinal failure, with no other associated diseases, and who do not tolerate parenteral nutrition. In fact, small intestine transplantation is the final option and it should only be used when all other common procedures fail and when it is expected that the patient will certainly develop intestinal and cholestatic liver failures.^4,5^

## CONCLUSIONS

Primary CIIPO is a heterogeneous disease and the main goals of intervention in such cases should be early diagnosis (to avoid repeated laparotomy), maintenance of good nutrition, symptom control and attention to motility recovery, which are all associated with long-term patient follow-up.
